# *Nrl:CreERT2* mouse model to induce mosaic gene expression in rod photoreceptors

**DOI:** 10.3389/fnmol.2023.1161127

**Published:** 2023-04-25

**Authors:** Molly T. Thorson, Stephanie E. Wei, Craig Johnson, Christopher J. Gabriel, Vadim Y. Arshavsky, Jillian N. Pearring

**Affiliations:** ^1^Department of Ophthalmology and Visual Sciences, University of Michigan, Ann Arbor, MI, United States; ^2^Department of Cell and Developmental Biology, University of Michigan, Ann Arbor, MI, United States; ^3^Department of Ophthalmology, Duke University, Durham, NC, United States; ^4^Department of Pharmacology and Cancer Biology, Duke University, Durham, NC, United States

**Keywords:** photoreceptor, outer segment, inducible, RD9, NRL, retinal degeneration, disc renewal

## Abstract

Photoreceptors are sensory neurons that capture light within their outer segment, a narrow cylindrical organelle stacked with disc-shaped membranes housing the visual pigment. Photoreceptors are the most abundant neurons in the retina and are tightly packed to maximize the capture of incoming light. As a result, it is challenging to visualize an individual cell within a crowded photoreceptor population. To address this limitation, we developed a rod-specific mouse model that expresses tamoxifen-inducible cre recombinase under the control of the *Nrl* promoter. We characterized this mouse using a farnyslated GFP (GFPf) reporter mouse and found mosaic rod expression throughout the retina. The number of GFPf-expressing rods stabilized within 3 days post tamoxifen injection. At that time, the GFPf reporter began to accumulate in basal disc membranes. Using this new reporter mouse, we attempted to quantify the time course of photoreceptor disc renewal in WT and Rd9 mice, a model of X-linked retinitis pigmentosa previously proposed to have a reduced disc renewal rate. We measured GFPf accumulation in individual outer segments at 3 and 6 days post-induction and found that basal accumulation of the GFPf reporter was unchanged between WT and Rd9 mice. However, rates of renewal based on the GFPf measurements were inconsistent with historical calculations from radiolabeled pulse-chase experiments. By extending GFPf reporter accumulation to 10 and 13 days we found that this reporter had an unexpected distribution pattern that preferentially labeled the basal region of the outer segment. For these reasons the GFPf reporter cannot be used for measuring rates of disc renewal. Therefore, we used an alternative method that labels newly forming discs with a fluorescent dye to measure disc renewal rates directly in the Rd9 model and found it was not significantly different from WT. Our study finds that the Rd9 mouse has normal rates of disc renewal and introduces a novel *Nrl:CreERT2* mouse for gene manipulation of individual rods.

## Introduction

The retina is a layered tissue designed to generate light-induced electrical responses that are ultimately sent to the brain for visual perception. The light-sensitive photoreceptors are the most abundant retinal neurons, which are tightly packed to ensure that the maximal number of photons passing through are detected. Photoreceptors are thin, highly compartmentalized neurons containing four distinct compartments: the outer segment, the inner segment, the nuclear region, and the synaptic terminal. Each of these regions can be collectively recognized as a discrete layer; however, it is challenging to identify an individual photoreceptor within the crowded population.

Studying cellular processes such as intracellular trafficking, release of neurotransmitters, or outer segment biogenesis in photoreceptors is important for understanding how vision works on the cellular level and can inform disease pathogenesis of inherited retinal dystrophy that leads to human blindness. However, the dense packing of photoreceptors limits the investigation of these processes at the single cell level. We sought to develop a mouse model that targets a sparse number of photoreceptors for genetic manipulation and allows individual photoreceptors to be identified from their neighbors. We envision that the availability of such a model could be used to alter the expression of any gene of interest in a subset of the photoreceptor population while maintaining a larger population of untargeted rods to serve as an internal control. Furthermore, a multitude of floxed reporters could be combined with this model to visualize key cellular processes within rods, such as mitophagy, intracellular trafficking, or, as we have done here, outer segment renewal.

The rod outer segment is a narrow, cylindrical organelle filled with hundreds of flattened membranous discs that are packed with the photosensitive pigment, rhodopsin. Throughout the life of a photoreceptor, the outer segment exists in a constant state of renewal whereby new discs are continuously added to the outer segment base and old discs are phagocytosed from the outer segment tip by overlying retinal pigment epithelial cells. Pioneering studies in the 1960–70s used pulse-chase style radiolabel experiments to track the addition of discs in *Xenopus laevis* outer segments over time and showed new discs turnover at a rate of ~2.4 μm per day (Young, 1967; Besharse et al., 1977). Quantitative estimates of the rate of disc turnover were possible in *Xenopus* photoreceptors due to their large size and single layer configuration. However, when this technique was applied to shorter mouse outer segments, it was challenging to determine precise disc renewal rate because the radioactive band was too diffuse to measure small fluctuations over brief intervals, although it was estimated to turnover at a rate of ~2.3 μm per day (Young, 1967; Besharse and Hollyfield, 1979). The mouse retina produces a diffuse band because the bases of individual outer segments are not aligned laterally. The ability to measure outer segment assembly in mice is imperative as they provide an unmatched number of retinal degenerative models that could accelerate our understanding of disease pathogenesis.

Here, we created a rod-specific inducible mouse model that drives CreERT2 expression from the *Nrl* promoter. Breeding this mouse with a lox-stop-lox, membrane-associated, farnesylated GFP (GFPf) reporter mouse resulted in excision of the stop codon and subsequent expression of GFPf only in a subset of rod cells. We found the GFPf reporter accumulated in the basal outer segment and attempted to track reporter accumulation to measure outer segment renewal over time in WT and a model of X-linked retinitis pigmentosa, Rd9. The Rd9 mouse contains a naturally occurring, 32 bp duplication within exon 15 of the retinitis pigmentosa GTPase regulator (*Rpgr*) gene (Thompson et al., 2012). This frameshift mutation results in early termination and complete loss of the photoreceptor-specific RPGR^ORF15^ isoform, but preserves normal expression of the ubiquitous RPGR^default^ isoform. Importantly, mutations within exon 15, encoding for the RPGR^ORF15^ isoform, are a hotspot for human retinal disease (Vervoort et al., 2000; Breuer et al., 2002; Sharon et al., 2003). It was previously shown that Rd9 mice have shortened outer segment length (Thompson et al., 2012), which could indicate that new disc formation is slowed. Additionally, RPGR was shown to interact with the actin severing protein, gelsolin, and hypothesized to regulate actin disassembly in the outer segment (Megaw et al., 2017, 2022), a process involved in nascent disc formation (Spencer et al., 2020). We measured GFPf accumulation in WT and Rd9 on the *Nrl:CreERT2/GFPf* background over time and found that the GFPf reporter is diffusing across disc membranes. Thus, it cannot be used to measure rates of disc renewal. However, using an alternative method we investigated the rate of disc renewal in a model of X-linked retinitis pigmentosa, Rd9, and report no changes in rates of disc synthesis.

## Materials and methods

### Animals

*Nrl:CreERT2* mice were generated at the Duke Transgenic Mouse Facility. Standard BAC Recombineering techniques were used to introduce an internal ribosome entry site (IRES), a tamoxifen-inducible CreERT2, and neomycin cassette behind the stop codon for *Nrl* within exon 4, preserving endogenous expression of Nrl. Neo selection was used to select positive embryonic stem (ES) cell clones that were then validated by both Long Range PCR and Southern blot. Validated ES clones were then injected into the 8-cell embryo stage, using the VelociMouse method as described in DeChiara et al. (2010), to generate *Nrl:CreERT2* chimeras. Founders were sequence verified and positive mice backcrossed to *C57Bl6/J* for 3–5 generations to generate the *Nrl:CreERT2* line. Mice were maintained as *C57Bl6/J* congenics. *RPGR^Rd9^* mice (RRID:MGI:3720014) were obtained from Dr. Debra Thompson at the University of Michigan (Thompson et al., 2012). *Gt(ROSA)26Sor^tm1(CAG-EGFP)Blh^* (RRID:MGI:3850368; *Rosa26^fGFP^*) mice were obtained from Dr. Jeremy Kay at Duke University (Rawlins et al., 2009; Ray et al., 2018). iCre75 mice (RRID:IMSR_JAX:015850) were obtained from Dr. Nina Haider from the Schepens Eye Institute (Li et al., 2005).

Mice were handled following protocols approved by the Institutional Animal Care and Use Committees at Duke University (registry number A011-14-01) and the University of Michigan (registry number A3114-01). As there are no known gender-specific differences related to vertebrate photoreceptor development and/or function male and female mice were randomly allocated to experimental groups. All mice were housed in a 12/12-h light/dark cycle with free access to food and water.

Genotyping: For genotyping, toe or tail clips were digested with DirectPCR Lysis Reagent (Viagen Biotech, 102-T) supplemented with 5% Proteinase K overnight at 55°C. Proteinase K is deactivated at 85°C for 30 min and debris spun down at ~13,000 rpm for 10 min. One microliter of crude lysate is used in a genotyping reaction performed with NrlCre-F (ACCTCTATAAGGCCCGCTGT), NrlCre-R1 (CGCCTTTGCAGGTGTATCTT), and NrlCre-R2 (AGGGAACTCATCTCCAGCAA), which produces a 264 bp product in WT and a 421 bp product in the *Nrl:CreERT2* allele. To genotype Rd9 mice, tissue was digested and DNA purified using Monarch^®^ Genomic DNA Purification Kit (New England BioLabs, T3010L). A ~ 2 kb DNA product was PCR amplified using Rd9-F1 (ACACCGTGCACTGAAAATGA) and ORF15 R1476 (TGTGCCATGTCTGCCATATT) and sent for Sanger Sequencing with Rd9-seq-F1 (AGGATGGGAGAAAGGGAGTG). Both alleles are then compared at the *RPGR* locus to the reference mouse genome GRCm39. A 32 bp duplication present in one or both alleles is verified to identify heterozygous or homozygous animals, respectively.

Tamoxifen Injections: Tamoxifen was mixed at a concentration of 20 μg/μL with Mazola corn oil and placed in a sonicating water bath until dissolved and injected intraperitoneally at a concentration of 150 mg/kg body weight.

Intraocular Injections: 1 mg of *CF*-568 hydrazide (Sigma-Aldrich, SCJ4600025) was reconstituted in 100 μL of DPBS to make a 1% solution. One microliter of 0.1% *CF*-568 was injected intravitreally into both eyes of adult mice. Five days post-injection WT and Rd9 mice were sacrificed, enucleated, and retinas collected for outer segment isolation. Fresh retinas were collected in 225 μL of Mouse Ringers, at pH 7.4 and ~ 313–320 mOsM (130 mM NaCl, 3.6 mM KCl, 2.4 mM MgCl_2_, 1.2 mM CaCl_2_, 10 mM HEPES, 0.02 mM EDTA), vortexed for 1 min on high, and spun down using a benchtop spinner for 5 s. ~100 μL of the supernatant was plated on poly-lysine coated #1.5 glass-bottom wells (Fisher Scientific, NC0704855) for live imaging. To quantify *CF*-568 band displacement, measurements were taken between the outer segment base, identified by the presence of a connecting cilium in DIC images, and the distal edge of the *CF*-568 fluorescent band. Outer segments that did not show a clear *CF*-568 band, had poor morphology, or did not have a discernible ciliary base were excluded from analysis.

### Immunoblot

Eyecups were homogenized *via* onication in 250 μL of PBS with Complete Protease Inhibitor (CPI, Millipore Sigma, 11836170001). Lysates were spun at 60,000 rpm for 20 min at 4°C. The supernatant was collected as the soluble fraction and the pellet was resuspended in 250 μL of PBS with 20% SDS and CPI and collected as the membrane fraction. The membrane fraction was subsequently spun at 14,000 rpm to pellet any remaining cellular debris. AnyKd Mini-PROTEAN TGX Precast Protein Gels (Bio-Rad, 4569033) were loaded with 30 μL of each lysate and SDS-PAGE gel run at 55 V for 30 min and then 120 V until the dye front ran to the bottom. This was followed by transfer at 90 mV for 90 min onto Immun-Blot Low Fluorescence PVDF Membrane (Bio-Rad, 1620264). Membranes were blocked using Intercept Blocking Buffer (LI-COR Biosciences, 927-70003). Antibodies used for Western blotting were mouse monoclonal anti-GFP (1:1,000, Takara Bio Cat# 632380, RRID:AB_10013427) and donkey anti-mouse IRDye 680RD (LI-COR Biosciences Cat# 926–68,072, RRID:AB_10953628).

### Immunofluorescence

Retinal cross-sections: Eyes were collected and drop-fixed for 1–2 h in 4% paraformaldehyde in PBS, rinsed three times in PBS, and dissected into posterior eyecups. Eyecups were embedded in 4.0% agarose (Fisher BioReagents, BP160-500) and 100 μm sections through the central retina were collected using a Leica VT1200s vibratome. Floating retinal sections were blocked for 1 h in 5% donkey serum with 0.5% TritonX-100 then incubated in primary antibody overnight. The following day, sections were rinsed three times with PBS and incubated in secondary antibody and fluorescent-conjugated markers for 2 h. Sections were then rinsed three times in PBS and mounted with Prolong Glass (Thermo Fisher Scientific, P36980) under High Precision #1.5 coverslips (Bioscience Tools, CSHP-No1.5-22 × 22). Antibodies and markers used: anti-GFP (1:2,000; Dr. Dawen Cai, University of Michigan), Wheat Germ Agglutinin (1:2000, WGA; Thermo Fisher Scientific, W32464), Hoechst (1:1,000; Fisher Scientific, BDB561908), donkey anti-chicken Alexa Fluor 488 (1:1000, Jackson ImmunoResearch Labs Cat# 703–546-155, RRID:AB_2340376).

Retinal whole-mounts: Eyes were collected and drop-fixed for 1–2 h in 4% paraformaldehyde in PBS, rinsed three times in PBS, and retinas dissected away from posterior eyecup. Retinas were blocked overnight in 5% donkey serum with 0.5% TritonX-100 and incubated in anti-GFP antibody (1:1000) with WGA (1:1000) for 3–4 days. Retinas were rinsed three times with PBS and incubated overnight in 4′,6-Diamidine-2′-phenylindole dihydrochloride (1:1000, DAPI; Sigma-Aldrich, 10236276001) and donkey anti-chicken Alexa Fluor 488 (1:1000). For mounting, the retina was flattened by cutting into 4 petals and the photoreceptor side of the retina was placed toward the coverslip (poly-L-lysine coated #1.5; Electron Microscopy, 50-192-9541) and mounted with Prolong Glass (Thermo Fisher Scientific, P36980).

Images were acquired using a Zeiss Observer 7 inverted microscope equipped with a 63x oil-immersion objective (1.40 NA) and LSM 800 confocal scanhead controlled by Zen 5.0 software (Zeiss).

### Image analysis

Outer segment length was quantified using the Imaris 9.5.64 visualization software. The Surfaces module was used to create 3D surface reconstructions of the green fluorescence within the outer segment compartment. The intensity threshold was determined manually and applied consistently to all images from the same staining conditions. Any surfaces that were inadvertently drawn in the inner segment were excluded from the length quantification. Length was measured using the filament module in Imaris to draw a dendrite with a manually defined beginning and endpoint within the 3D surface. Dendrite length was exported to Microsoft Excel for further analysis.

### Statistical analysis

All data represent at least three independent animals or experiments. For rate of rod induction after a single dose of tamoxifen at P24 (Figure 3), a one-way ANOVA test was performed using Prism 9 software (Graphpad). For comparing GFPf accumulation lengths (Figure 4), a fitting linear mixed-effects model was used and implemented through the lme4 package (v1.1–30) of the R statistical language version 4.1.2 (Bates et al., 2015). A cell means version of the model was fit with animal as a random effect and looked like lengths ~0 + Genotype_Time + (1|Subject). General linear hypothesis testing was carried out with the glht function of multcomp R package (v1.4–19) (Hothorn et al., 2008). For *CF*-568 displacement in WT versus RD9 mice (Figure 5), a student *t*-test was performed on the average of averages from *n* = 12 using Prism 9. Throughout the figures the data is presented as a mean with ± standard deviation and corresponding *p-*values are listed in the figure legends.

**Figure 1:**
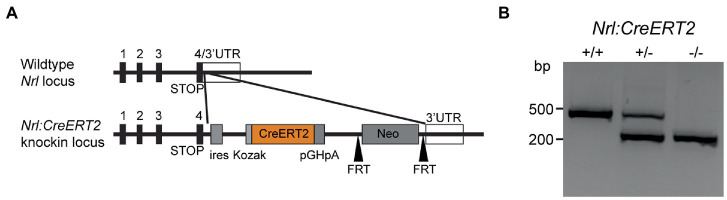
Tamoxifen-inducible *Nrl:CreERT2* mouse. **(A)** Gene diagram for *CreERT2* insertion within the *Nrl* locus. **(B)** Agarose gel showing bands produced by genotyping PCRs on isolated genomic DNA from *Nrl:CreERT2^+/+^*, *Nrl:CreERT2^+/−^*, and *Nrl:CreERT2^−/−^* mice.

**Figure 2:**
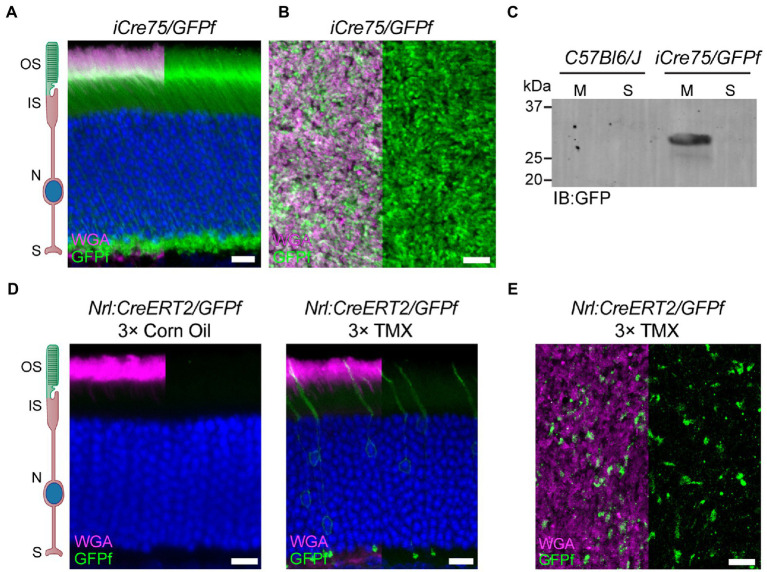
*Nrl:CreERT2* mouse displays a mosaic pattern of reporter gene expression in rod photoreceptors. **(A)** Representative retinal cross-section from an *iCre75/GFPf* mouse stained with anti-GFP antibody and WGA. Nuclei are counterstained with Hoechst. **(B)** Representative image of a retinal whole-mount from an *iCre75/GFPf* mouse stained with anti-GFP antibody and WGA. **(C)** Membrane and soluble retinal fractions from *C57Bl6/J* and *iCre75/GFPf* mice immunoblotted (IB) for GFP. **(D,E)** *Nrl:CreERT2/GFPf* mice were injected 3 consecutive days with either tamoxifen or corn oil. Eyes were collected 2 weeks post-injection. **(D)** Retinal cross-sections stained with anti-GFP antibody and WGA. Nuclei are counterstained with Hoechst. **(E)** Retinal whole-mount stained with anti-GFP antibody and WGA. Scale Bars, 10 μm. Here and in all figures: outer segment (OS), inner segment (IS), nuclei (N), and synapse (S).

**Figure 3:**
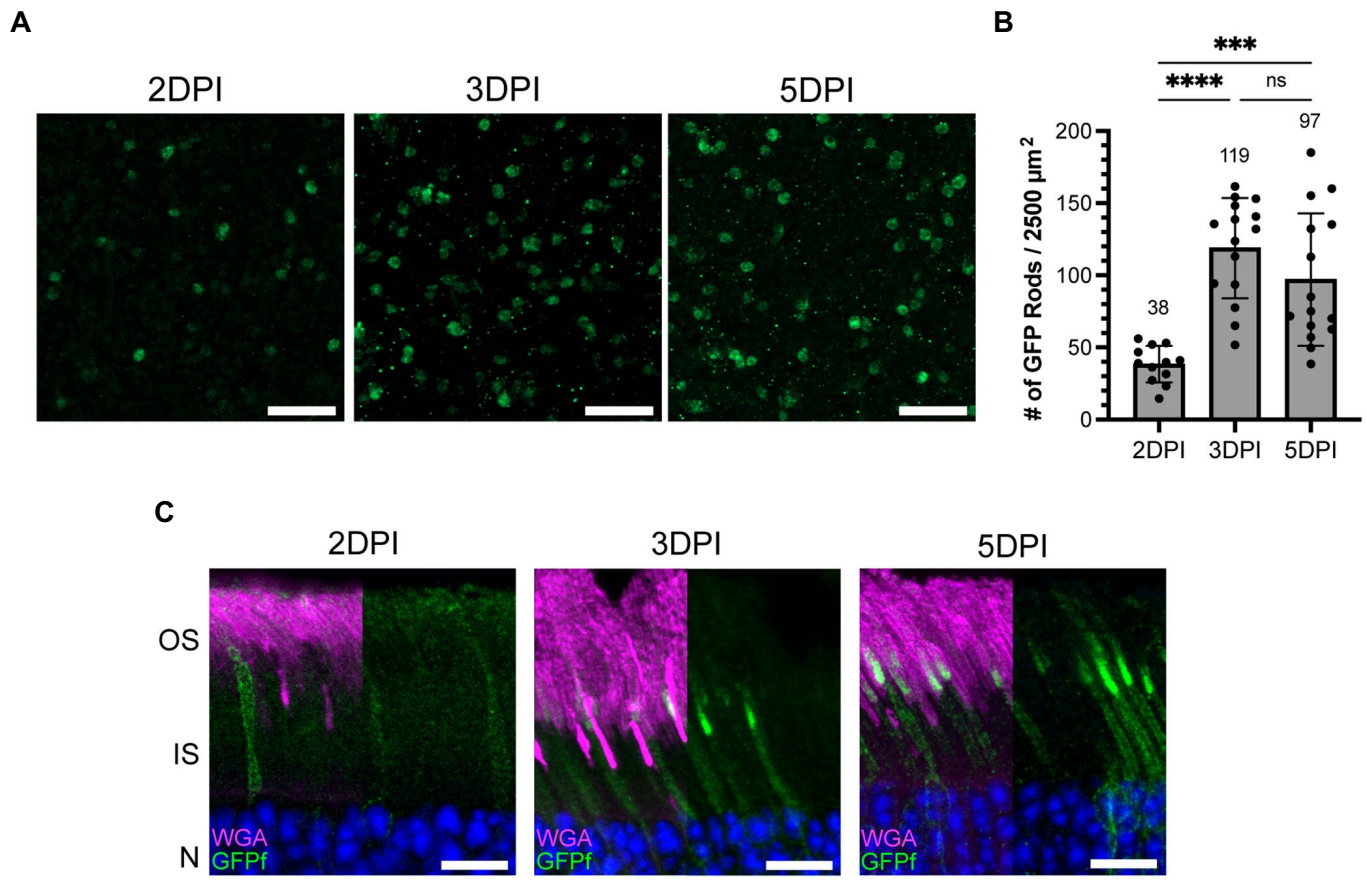
The number of rod photoreceptors expressing GFPf in the *Nrl:CreERT2* mouse stabilizes 3 days post tamoxifen induction. **(A)** Representative retinal whole mounts from *Nrl:CreERT2/GFPf* stained with anti-GFP antibody showing GFP expression in the rod synapse at 2, 3, and 5 days post-injection (DPI). Scale Bars, 10 μm. **(B)** Bar graph plotting the number of GFP positive rods in a 2,500 μm^2^ retinal area. Mean for each timepoint is displayed above the bar. Error bars represent S.D. A one-way ANOVA was preformed: ****p < 0.0001, ***p = 0.0003, ns *p* = 0.2172. **(C)** Representative retinal cross-sections from *Nrl:CreERT2/GFPf* mice at 2, 3, and 5 DPI stained with anti-GFP antibody and WGA. Nuclei are counterstained with Hoechst. Scale Bars, 10 μm.

**Figure 4:**
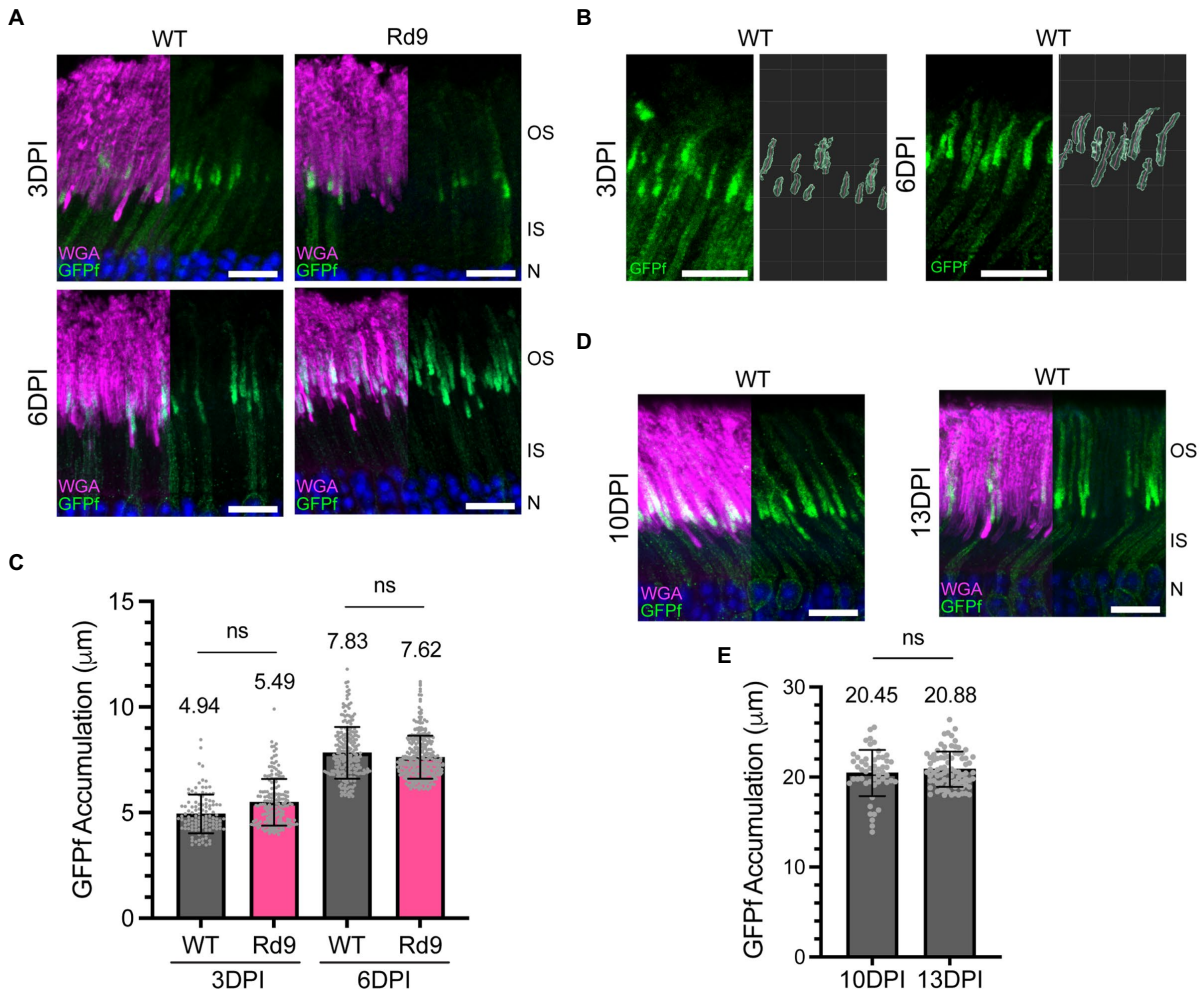
Investigating whether the *Nrl:CreERT2/GFPf* model can be used to measure rates of outer segment renewal. **(A)** Retinal cross-sections from WT and Rd9 mice on the *Nrl:CreERT2/GFPf* background at 3 and 6 DPI stained with anti-GFP antibody and WGA. Nuclei are counterstained with Hoechst. Scale bars, 10 μm. **(B)** Representative images showing the analysis used to measure the length of GFPf accumulation in outer segments. **(C)** Bar graph plotting stratified measurements of GFPf accumulation in outer segments. Mean values are displayed above the bars. Error bars represent S.D. Linear mixed modeling: 3 DPI ns *p* = 0.531 and 6 DPI ns *p* = 0.896. **(D)** Retinal cross-sections from WT mice on the *Nrl:CreERT2/GFPf* background at 10 and 13 DPI stained with anti-GFP antibody and WGA. Nuclei are counterstained with Hoechst. Scale bars, 10 μm. **(E)** Bar graph plotting stratified measurements of GFPf accumulation in outer segments. Mean values are displayed above the bars. Error bars represent S.D. Linear mixed modeling: ns *p* = 0.704.

**Figure 5:**
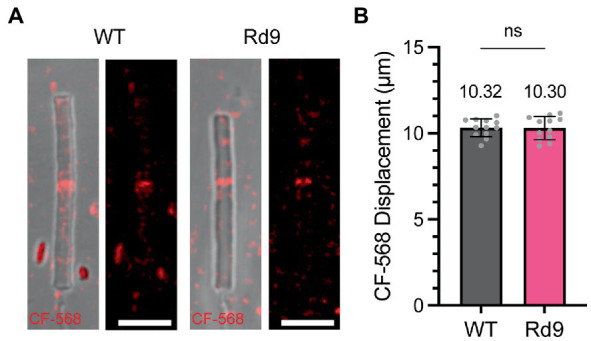
Rate of new disc addition is not affected by loss of RPGR-ORF15. **(A)** Images showing representative isolated outer segments from WT and Rd9 mice 5 days after *CF*-568 injection. Differential interference contrast (DIC) overlay on left and *CF*-568 alone on right. Scale bar, 5 μm. **(B)** Bar graph plotting the distance between the outer segment base and the band of incorporated *CF*-568. Mean values are displayed above the bar. Error bars represent S.D. Unpaired Student’s two-tailed *t-*test: ns *p* = 0.9543.

## Results

### Generation of the *Nrl:CreERT2* mouse model for mosaic gene expression in rod photoreceptors

We targeted the neural retina leucine zipper (*Nrl*) locus that encodes a transcription factor regulating rod photoreceptor cell fate (Swaroop et al., 1992; Swain et al., 2001; Coolen et al., 2005). Our targeting vector included an internal ribosome entry site (IRES), a tamoxifen-inducible CreERT2, and neomycin cassette, which was inserted behind the stop codon for *Nrl* within exon 4 to preserve endogenous expression of Nrl (Figure 1A). Founders were screened for proper insertion using long-arm PCR and southern blotting (data not shown) and positive mice then backcrossed to *C57Bl6/J* for 3–5 generations to generate the *Nrl:CreERT2* line. Genotyping was designed to differentiate between WT and *Nrl:CreERT2* knock-in alleles (Figure 1B).

To characterize the expression of CreERT2 in our mouse, we crossed it with a mouse line bearing a floxed, fluorescent reporter. Because the outer segment has a membrane-rich architecture that restricts accessibility of soluble proteins, including fluorophores (reviewed in Barnes et al., 2021), we used an untargeted, membrane-bound reporter that would label the entire rod photoreceptor, from synapse to outer segment (Baker et al., 2008). We chose the *Rosa26f^GFP^* reporter mouse that contains a lox-stop-lox, farnesylated GFP at the *Rosa26* locus (referred to as *GFPf*). Initially, we characterized GFPf localization in rod photoreceptors by crossing the *GFPf* reporter mouse to a common transgenic mouse model for rod-specific expression of cre recombinase, *iCre75* (Li et al., 2005). Retinal cross-sections from the *iCre75/GFPf* mouse showed GFPf expression throughout the entirety of all expressing rods (Figures 2A,B). We then confirmed membrane-association of GFPf by collecting membrane and soluble fractions from retinal lysates of *iCre75/GFPf* and *C57Bl6/J* mice at P30. The GFPf band was only present in the membrane fraction of *iCre75/GFPf* lysates (Figure 2C).

We continued to use the GFPf reporter to characterize the tamoxifen induction of CreERT2 in our *Nrl:CreERT2* mouse. Induction efficiency was analyzed by treating mice at 6 weeks of age with three intraperitoneal (IP) injections of either tamoxifen or a corn oil sham control. Mice were then sacrificed 2 weeks later and eyes collected for cross-section or whole-mount staining. Tamoxifen injections resulted in cre recombination and GFPf expression in a sparse amount of rod photoreceptors (Figures 2D,E). The mosaic expression of GFPf in rods occurred throughout the entire retina (Supplementary Figure 1).

### Number of GFPf expressing rods plateaus after induction

After generating the inducible mouse model, we set out to determine the rate at which cre recombination occurs following tamoxifen injection. Using three subsequent injections, as done in Figure 2, would confound our ability to accurately determine when tamoxifen induced rod GFPf expression. Therefore, cre expression was induced by a single IP injection of tamoxifen at P24 in *Nrl:CreERT2/GFPf* mice and eyes were collected 2, 3, and 5 days post injection. The number of rod cells that express GFPf on a given day was determined by performing GFP immunostaining on whole-mount retinas (Figure 3A). We identified the outer plexiform layer within retinal whole mounts using an Alexa Fluor 594 conjugated WGA, a lectin that labels photoreceptor membranes, then imaged and counted GFP-expressing rod synapses. For each retina, the number of GFP-expressing rods was quantified from 5 different regions (2–4 images of a ~ 2,500 μm^2^ area were collected at each region; Figure 3B). From this analysis, we found that induction of CreERT2 gradually ramps up until 3 days post-injection when it then plateaus. Therefore, the rate of induction is approximately 3 days after a single tamoxifen injection. Since GFPf-expressing rods were equally distributed throughout the retina (see Supplementary Figure 1), we extrapolated these data to estimate the percentage of total rods that express GFPf and found that ~6–8% of rods were induced (Supplementary Figure 2). These data also demonstrate that a single IP injection of tamoxifen is sufficient for mosaic rod expression and is used as the standard operating procedure going forward.

### GFPf accumulates in rod outer segment membranes after induction

By analyzing GFPf localization in retinal cross-sections at the same timepoints, we found that by 2 days post-injection GFPf was present in the plasma membrane surrounding the inner segment and cell body but was largely excluded from the outer segment. By 3 days post-injection, GFPf began to accumulate in the outer segment membranes at the base and by 5 days post-injection this accumulation had visibly increased in length (Figure 3C). Since the membrane-bound GFPf initially labeled the outer segment base and accumulated more apically with time it appeared that the GFPf reporter was stably bound to disc membranes. Therefore, we hypothesized that we could utilize the GFPf reporter to measure ongoing disc addition to investigate outer segment renewal in a model of retinal degeneration.

### *Nrl:CreERT2/GFPf* cannot be used to measure rates of disc renewal

Ongoing disc renewal is critically important to maintain outer segment structure and function, and defects in this process underly many forms of inherited retinal degeneration. We chose to investigate outer segment turnover in the Rd9 mouse that lacks the photoreceptor-specific RPGR^ORF15^ isoform, a molecule reported to play a role in disc renewal (Megaw et al., 2022). To test whether the rate of new disc addition is altered by the loss of RPGR^ORF15^, the *Nrl:CreERT2/GFPf* mice were bred to Rd9 mice, injected with tamoxifen at P24, and analyzed 3 and 6 days post-injection alongside WT littermate controls. Figure 4A shows representative retinal cross-sections stained with anti-GFP antibody and WGA to mark the outer segments. To quantitatively measure GFPf accumulation in outer segments of individual rods, we developed a semi-automatic analysis pipeline using Imaris software to construct 3D surfaces of GFPf signal. The surfaces were drawn based upon an intensity threshold that was applied consistently across images from the same staining conditions. The surfaces were then used as boundary markers to measure the outer segment length by manually selecting the outer segment base as a beginning point and the distal edge of GFPf fluorescence as the ending point. The filament algorithm of Imaris then defined the path between the manually drawn points through the center of fluorescence (Figure 4B).

Our analysis revealed unexpectedly low estimates of new disc addition even for WT mice (Supplementary Figure 3). We attributed this discrepancy to the delayed induction rate of GFPf expression shown in Figure 3. Based on this understanding, we assumed that the earliest population of GFPf-expressing rods represented only 30% of the total GFPf-expressing rods measured at subsequent days post-injection. Therefore, we only included in our analysis the top 30% of the measured outer segment lengths from each retina, as they represent the population of rods in which the reporter was first induced. However, we found that the length of GFPf accumulation in WT and Rd9 mice was not significantly different at 3 or 6 days post-injection (Figure 4C). Based on these GFPf measurements, we calculated the rate of disc turnover to be ~1 μm/day in WT mice (see Table 1). Even though initial radiolabeled measurements were unable to quantify daily rates of disc renewal, these experiments did clearly demonstrate that the ~22 μm long outer segment turnover occurs in 10–12 days in mice. Therefore, our calculated rate is too low to be accurate and prevents interpretation of renewal rates in Rd9 mice.

**Table 1:** Summary table of outer segment growth measurements compared to historical calculations.

Days post-injection	Genotype	Length of GFPf accumulation (μm)	^A^Rate of renewal (μm/day)
3	WT	4.94 ± 0.17	–
	Rd9	5.45 ± 0.17	–
6	WT	7.83 ± 0.16	1.0
	Rd9	7.62 ± 0.11	0.7
Days post-injection	Genotype	Distance to 3H-Band (μm)	^B^Rate of renewal (μm/day)
1	WT	1.8 ± 0.09	–
4	WT	8.8 ± 0.18	2.3
5	WT	11.1 ± 0.14	2.3
9	WT	19.9 ± 0.27	2.3
Days post-injection	Genotype	CF-568 band displacement (μm)	^C^Rate of renewal (μm/day)
5	WT	10.32 ± 0.33	2.6
	Rd9	10.30 ± 0.43	2.6

Length of GFPf accumulation shown as a 95% confidence interval around the mean. ^A^Rate of renewal calculated with the formula: (L_t6_ – L_t3_)/(t_6_ – t_3_), where L is length of GFPf accumulation and t_3_ is 3 days post-injection and t_6_ is 6 days post-injection. Original data that uses 3H-Band displacement in WT mice to calculate rates of disc renewal were taken from (Besharse and Hollyfield, 1979). ^B^Rate of renewal calculated with the formula: (B_t2_ – B_t1_)/(t_2_ – t_1_), where B is 3H-band position and t_1_ is 1 day post-injection and t_2_ is a subsequent day. CF-568 band displacement shown as a 95% confidence interval around the mean. ^C^Rate of renewal calculated with the formula: B_t5_/(t_5_ – 1), where B is CF-568 band position and t_5_ is 5 days post-injection.

An insight into why the GFPf reporter was not performing as expected came when we allowed GFPf to accumulate in the outer segment for an entire turnover cycle. Figure 4D shows that GFPf labeled the entire outer segment but was not equally distributed. Rather, it was highly enriched at the outer segment base. The same localization pattern of GFPf within the outer segment was also observed at 13 days post injection (Figure 4D). Also, the measured lengths between 10 and 13 days post-induction were not significantly different and were consistent with full length outer segments in mice (Figure 4E; LaVail, 1973). This weighted localization pattern was surprising because we expected that, if GFPf were stably bound to disc membranes, complete outer segment turnover would result in an equal intensity of GFPf throughout the entire outer segment. These observations are not consistent with the GFPf being stably bound to disc membranes, which essentially rejects the utility of GFPf to be used as a tool for tracking the rate of outer segment renewal. Further explanation regarding our insight into the underperformance of the GFPf reporter can be found in the discussion.

### Rd9 mice show no change in the rate of outer segment renewal

To investigate outer segment renewal rates in WT and Rd9 mice, we used a method recently developed by Reed and colleagues that tracks the displacement of the water-soluble, inert dye *CF*-568 after intraocular injection (Reed et al., 2022). *CF*-568 was shown to incorporate into nascent basal discs that are exposed to the extracellular environment. In rods, nascent discs are enclosed trapping the dye in the intradiscal space and creating a *CF*-568 band that travels distally as more discs are added to the outer segment base. Displacement of the band over time represents the rate of new disc addition. WT and Rd9 mice were injected intraocularly with *CF*-568 and 5 days later outer segments were isolated, live-imaged, and *CF*-568 band displacement from the base was measured. Figure 5A shows representative images of isolated outer segments from WT and Rd9 mice labeled with *CF*-568. Importantly, we found that the distance between the outer segment base and the *CF*-568 band was ~10.3 μm for both WT and Rd9 animals (Figure 5B). From these measurements, we calculate the rate of disc renewal to be ~2.6 μm/day in both models (see Table 1), which is consistent with previously reported rates of disc synthesis and confirms that our measurements with GFPf are lower than expected. We conclude that the loss of RPGR^ORF15^ does not impact the rate of disc morphogenesis.

## Discussion

Using the *Nrl* locus we developed a new mouse model for inducible, rod-specific expression of floxed genes. We characterized the gene expression pattern in this mouse using a farnesylated GFP reporter and found that a single tamoxifen injection produced mosaic rod-expression that was complete by 3 days post injection. Additionally, it was observed that the number of tamoxifen injections did not affect the final number of rods expressing GFPf. We also found that outer segment accumulation of the membrane-associated GFPf reporter increased with time until the entire compartment was labeled by 10 days. When compared to the *iCre75/GFPf* mouse, in which individual photoreceptors are indecipherable, the *Nrl:CreERT2/GFPf* mouse allows individual cells to be delineated within the dense population.

We hypothesized that the single cell resolution provided by this model could be used to monitor the ongoing addition of new disc membranes over time and attempted to investigate outer segment renewal rates in a model of retinal degeneration. We focused on the Rd9 mouse, a model of RPGR-associated X-linked retinitis pigmentosa, because these mice have shorter outer segments and a previous study suggested RPGR regulates actin dynamics at the outer segment base, further implicating it in outer segment renewal (Thompson et al., 2012; Megaw et al., 2017). Our initial approach to analyzing new disc synthesis in the outer segment using the *Nrl:CreERT2* mouse seemed promising; however, the GFPf reporter failed to serve as a proxy for outer segment disc synthesis.

An explanation for this unexpected result could be found in a recent study demonstrating that lipidated fluorescent reporters have a relatively weak membrane affinity and can diffuse throughout the outer segment at a rate significantly faster than the rate of disc renewal (Maza et al., 2019). Because our 3 and 6 day images showed predominant basal labeling of the outer segment, we assumed that GFPf was not behaving as a diffusible molecule. Otherwise, the fluorophore would be expected to label the entire outer segment in an equal distribution, even at early timepoints. However, Maza and colleagues further observed that GFP reporters containing various lipid modifications can display different distribution patterns in the outer segment. For example, a myristoylated GFP reporter expressed in frog rods was distributed evenly throughout the outer segment length; however, a similar farnesylated GFP reporter was preferentially localized to the basal region of the outer segment (Maza et al., 2019). In our study, GFPf showed the same preferential localization to the outer segment base, which was clearly observed when expression levels were sufficient to label the entire outer segment compartment such as in retinal sections from *Nrl:CreERT2/GFPf* mice after 10–13 days post tamoxifen injection and *iCre75/GFPf* mice (Figures 2A, 4D). Our results are consistent with GFPf diffusing across membranes; however, they also argue that GFPf has differential affinity to membranes located at the base rather than the tip of the outer segment, which skews its distribution pattern. The reasons for this uneven affinity are not known and elucidating their nature is an exciting area of future investigations.

We went on to investigate the rates of outer segment renewal in WT and Rd9 mice using an alternative approach that measures displacement of nascent outer segment discs labeled with a fluorescent dye. We report the rate of disc synthesis to be ~2.6 μm/day, which was not different between WT and Rd9 mice and was consistent with previously published rates using the same method (Reed et al., 2022). The decreased outer segment length observed the Rd9 mouse could be explained by either alterations in disc shedding or in determinants setting the outer segment length during retinal development that remain poorly understood.

Our data demonstrate that loss of RPGR^ORF15^ does not impact ongoing disc synthesis in mouse rod photoreceptors. In contrast, a recent study using pulse-chase click chemistry of SNAP-tagged rhodopsin reported that a mouse model with loss of both RPGR isoforms, RPGR^ORF15^ and RPGR^default^, had reduced rates of renewal (Megaw et al., 2022). The authors found that, after 72 h, newly synthesized discs reached lengths of ~15 μm in WT mice. Based on these values, the rate of disc renewal in WT mice would be ~5 μm/day and accordingly, result in outer segment turnover occurring within ~4–5 days. This rate is surprisingly high compared to our data and all previous publications (Young, 1967; LaVail, 1973; Besharse and Hollyfield, 1979; Reed et al., 2022). Considering this large discrepancy between calculated rates, it would be difficult to draw conclusions from measurements collected in this manner on the RPGR knockout mouse. A possible explanation for the inflated rate is that measurements of SNAP-tagged rhodopsin were acquired from a densely labeled population of rods. Several factors can influence measurements acquired from compound staining including quality of cross-section, area within the retina, and depth of plane. Most importantly, these factors are exacerbated in mouse tissue where the bases of individual outer segments are not tightly aligned and visualizing individual outer segment bases from their neighbors is nearly impossible. Therefore, it is plausible that the width of the band representing newly formed discs in Megaw et al. (2022) was measured from the base of the most proximally positioned outer segments to the distal edge of newly formed discs in the most apically positioned outer segments. Since the misalignment amongst the outer segment bases extends up to ~7–8 μm, the length of the newly formed disc stacks produced within 3 days may be overestimated by a factor of ~2. Our *Nrl:CreERT2* mouse model circumvents these challenges by providing single rod resolution and clearly delineating individual outer segments within the crowded population.

In summary, our data show that the *Nrl:CreERT2* mouse provides exceptional capabilities to visualize and characterize individual rods. We believe that this new model is a valuable tool for future studies of photoreceptor cell biology and pathophysiology.

## Data availability statement

The original contributions presented in the study are included in the article/Supplementary material, further inquiries can be directed to the corresponding author.

## Ethics statement

The animal study was reviewed and approved by IACUC Committee at Duke University (A011-14-01) IACUC Committee at University of Michigan (A3114-01).

## Author contributions

MT and JP conceived, designed the study, and organized the dataset. JP and VA designed, funded, and produced the *Nrl:CreERT2* mouse. MT, CG, and JP performed mouse experiments. MT, SW, CG, and JP collected imaging data. MT and SW implemented imaging analysis pipeline. MT collected immunoblot data. MT and CJ performed the statistical analyses. MT and JP wrote the manuscript with input from VA. All authors read and approved the submitted version.

## Funding

This work was supported by NIH K12 grant GM111725 (MT), NIH grants EY032491 (JP), EY012859 (VA), NIH Core Grants EY007003 and EY005722, Matilda E. Ziegler Research Award (JP), Career Development Award from Research to Prevent Blindness (JP), and Unrestricted Grants to Duke University and the University of Michigan from Research to Prevent Blindness.

## Conflict of interest

The authors declare that the research was conducted in the absence of any commercial or financial relationships that could be construed as a potential conflict of interest.

## Publisher’s note

All claims expressed in this article are solely those of the authors and do not necessarily represent those of their affiliated organizations, or those of the publisher, the editors and the reviewers. Any product that may be evaluated in this article, or claim that may be made by its manufacturer, is not guaranteed or endorsed by the publisher.

## References

[ref1] Baker S. A. Haeri M. Yoo P. Gospe S. M. Skiba N. P. Knox B. E.. (2008). The outer segment serves as a default destination for the trafficking of membrane proteins in photoreceptors. J. Cell Biol. 183, 485–498. doi: 10.1083/jcb.200806009, PMID: 18981232 PMC2575789

[ref2] Barnes C. L. Malhotra H. Calvert P. D. (2021). Compartmentalization of photoreceptor sensory cilia. Front. Cell Dev. Biol. 9:636737. doi: 10.3389/fcell.2021.636737 33614665 PMC7889997

[ref3] Bates D. Machler M. Bolker B. Walker S. (2015). Fitting linear mixed-effects models using lme4. J. Stat. Softw. 67, 1–48. doi: 10.18637/jss.v067.i01

[ref4] Besharse J. C. Hollyfield J. G. (1979). Turnover of mouse photoreceptor outer segments in constant light and darkness. Invest. Ophthalmol. Vis. Sci. 18, 1019–1024. 478775

[ref5] Besharse J. C. Hollyfield J. G. Rayborn M. E. (1977). Turnover of rod photoreceptor outer segments. II. Membrane addition and loss in relationship to light. J. Cell Biol. 75, 507–527. doi: 10.1083/jcb.75.2.507, PMID: 264121 PMC2109927

[ref6] Breuer D. K. Yashar B. M. Filippova E. Hiriyanna S. Lyons R. H. Mears A. J.. (2002). A comprehensive mutation analysis of RP2 and RPGR in a North American cohort of families with X-linked retinitis pigmentosa. Am. J. Hum. Genet. 70, 1545–1554. doi: 10.1086/340848 11992260 PMC379141

[ref7] Coolen M. Sii-Felice K. Bronchain O. Mazabraud A. Bourrat F. Rétaux S.. (2005). Phylogenomic analysis and expression patterns of large Maf genes in Xenopus tropicalis provide new insights into the functional evolution of the gene family in osteichthyans. Dev. Genes Evol. 215, 327–339. doi: 10.1007/s00427-005-0476-y, PMID: 15759153

[ref8] DeChiara T. M. Poueymirou W. T. Auerbach W. Frendewey D. Yancopoulos G. D. Valenzuela D. M. (2010). Producing fully ES cell-derived mice from eight-cell stage embryo injections. Methods Enzymol. 476, 285–294. doi: 10.1016/S0076-6879(10)76016-X, PMID: 20691872

[ref9] Hothorn T. Bretz F. Westfall P. (2008). Simultaneous inference in general parametric models. Biom. J. 50, 346–363. doi: 10.1002/bimj.200810425 18481363

[ref10] LaVail M. M. (1973). Kinetics of rod outer segment renewal in the developing mouse retina. J. Cell Biol. 58, 650–661. doi: 10.1083/jcb.58.3.650, PMID: 4747920 PMC2109077

[ref11] Li S. Chen D. Sauvé Y. McCandless J. Chen Y. J. Chen C. K. (2005). Rhodopsin-iCre transgenic mouse line for Cre-mediated rod-specific gene targeting. Genesis 41, 73–80. doi: 10.1002/gene.20097, PMID: 15682388

[ref12] Maza N. A. Schiesser W. E. Calvert P. D. (2019). An intrinsic compartmentalization code for peripheral membrane proteins in photoreceptor neurons. J. Cell Biol. 218, 3753–3772. doi: 10.1083/jcb.201906024, PMID: 31594805 PMC6829649

[ref13] Megaw R. Abu-Arafeh H. Jungnickel M. Mellough C. Gurniak C. Witke W.. (2017). Gelsolin dysfunction causes photoreceptor loss in induced pluripotent cell and animal retinitis pigmentosa models. Nat. Commun. 8:271. doi: 10.1038/s41467-017-00111-8, PMID: 28814713 PMC5559447

[ref14] Megaw R. Moye A. Zhang Z. Newton F. McPhie F. Murphy L. C.. (2022). Ciliary tip actin dynamics regulate the cadence of photoreceptor disc formation. bioRxiv. doi: 10.1101/2022.11.10.516020 PMC11109262 38773095

[ref15] Rawlins E. L. Okubo T. Xue Y. Brass D. M. Auten R. L. Hasegawa H.. (2009). The role of Scgb1a1+ Clara cells in the long-term maintenance and repair of lung airway, but not alveolar, epithelium. Cell Stem Cell 4, 525–534. doi: 10.1016/j.stem.2009.04.002, PMID: 19497281 PMC2730729

[ref16] Ray T. A. Roy S. Kozlowski C. Wang J. Cafaro J. Hulbert S. W.. (2018). Formation of retinal direction-selective circuitry initiated by starburst amacrine cell homotypic contact. elife 7:e34241. doi: 10.7554/eLife.34241, PMID: 29611808 PMC5931800

[ref17] Reed M. Takemaru K. I. Ying G. Frederick J. M. Baehr W. (2022). Deletion of CEP164 in mouse photoreceptors post-ciliogenesis interrupts ciliary intraflagellar transport (IFT). PLoS Genet. 18:e1010154. doi: 10.1371/journal.pgen.1010154, PMID: 36074756 PMC9488791

[ref18] Sharon D. Sandberg M. A. Rabe V. W. Stillberger M. Dryja T. P. Berson E. L. (2003). RP2 and RPGR mutations and clinical correlations in patients with X-linked retinitis pigmentosa. Am. J. Hum. Genet. 73, 1131–1146. doi: 10.1086/379379, PMID: 14564670 PMC1180492

[ref19] Spencer W. J. Lewis T. R. Pearring J. N. Arshavsky V. Y. (2020). Photoreceptor discs: Built like ectosomes. Trends Cell Biol. 30, 904–915. doi: 10.1016/j.tcb.2020.08.005 32900570 PMC7584774

[ref20] Swain P. K. Hicks D. Mears A. J. Apel I. J. Smith J. E. John S. K.. (2001). Multiple phosphorylated isoforms of NRL are expressed in rod photoreceptors. J. Biol. Chem. 276, 36824–36830. doi: 10.1074/jbc.M105855200, PMID: 11477108

[ref21] Swaroop A. Xu J. Z. Pawar H. Jackson A. Skolnick C. Agarwal N. (1992). A conserved retina-specific gene encodes a basic motif/leucine zipper domain. Proc. Natl. Acad. Sci. U. S. A. 89, 266–270. doi: 10.1073/pnas.89.1.266, PMID: 1729696 PMC48217

[ref22] Thompson D. A. Khan N. W. Othman M. I. Chang B. Jia L. Grahek G.. (2012). Rd9 is a naturally occurring mouse model of a common form of retinitis pigmentosa caused by mutations in RPGR-ORF15. PLoS One 7:e35865. doi: 10.1371/journal.pone.0035865, PMID: 22563472 PMC3341386

[ref23] Vervoort R. Lennon A. Bird A. C. Tulloch B. Axton R. Miano M. G.. (2000). Mutational hot spot within a new RPGR exon in X-linked retinitis pigmentosa. Nat. Genet. 25, 462–466. doi: 10.1038/78182, PMID: 10932196

[ref24] Young R. W. (1967). The renewal of photoreceptor cell outer segments. J. Cell Biol. 33, 61–72. doi: 10.1083/jcb.33.1.61, PMID: 6033942 PMC2107286

