# Supplementary material for: Nrl:CreERT2 mouse model to induce mosaic gene expression in rod photoreceptors
Source: Front Mol Neurosci. 2023 Apr 25;16:1161127. doi: 10.3389/fnmol.2023.1161127 (PMC10166802; doi:10.3389/fnmol.2023.1161127)
Supplement: Supplementary file 1 [file Data_Sheet_1.PDF]

Supplementary Material

Nrl:CreERT2 mouse model to induce mosaic expression in rod photoreceptors

Molly T. Thorson[1], Stephanie E. Wei[1], Craig Johnson[4], Christopher J. Gabriel[2], Vadim Y. Arshavsky[2,3], and Jillian N. Pearring[1,4,*]

*** Correspondence:** Jillian Pearring: pearring@umich.edu

Supplementary Figures

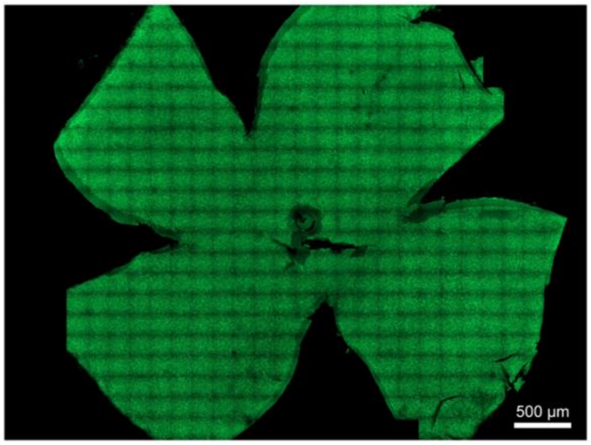

Supplementary Figure 1. Representative image of retinal whole mount from of *Nrl:CreERT2/GFPf* mouse stained with anti-GFP antibody (green) shows mosaic expression of GFPf in rods throughout the entire retina.

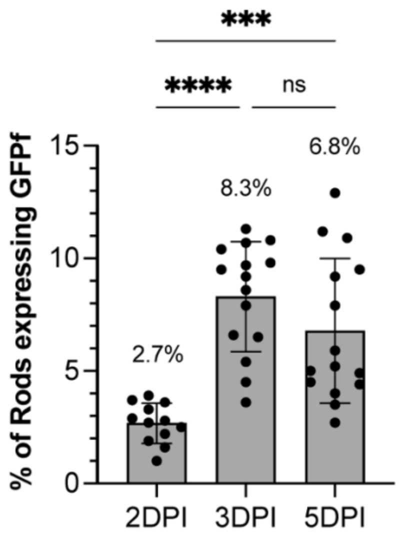

Supplementary Figure 2. Bar graph depicting the percent of rods in the retina that express GFPf after one dose of tamoxifen. The number of total rod cells within a 2500 µm^2 area was estimated by counting DAPI positive nuclei and averaging across the images collected for **Figure 3A**. The number of GFPf expressing rods from **Figure 3B** was then calculated as a percentage of the total rods. We interpret this as a representation of the % of GFPf expressing rods in the entire retina.

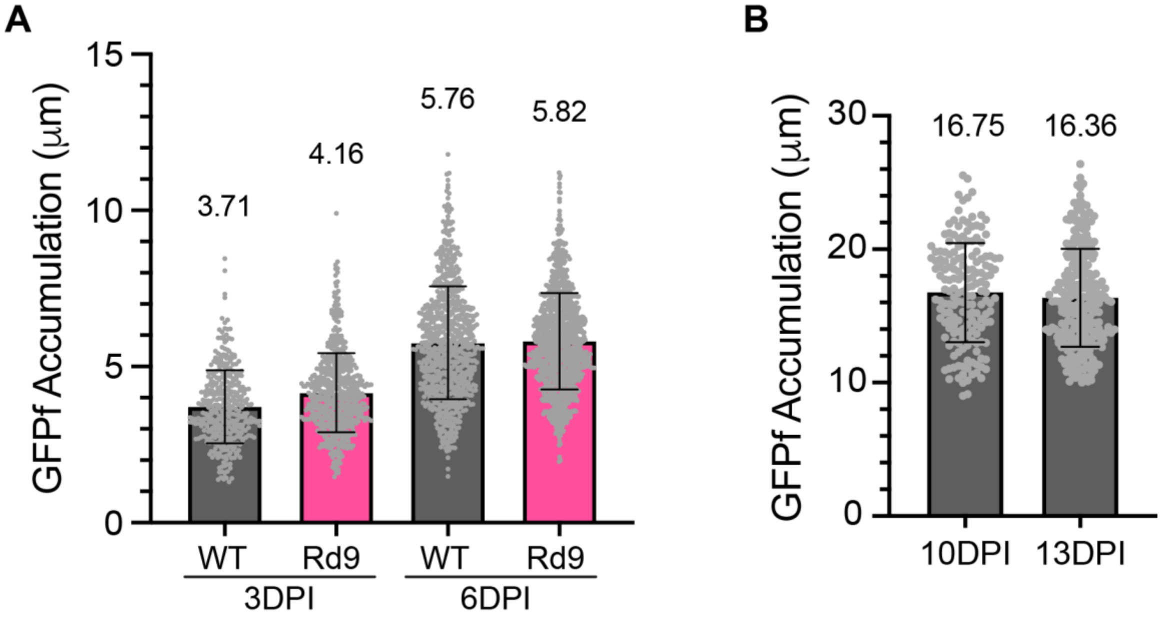

Supplementary Figure 3. Bar graphs showing a complete depiction of the GFPf measurement datasets for: (**A**) WT and Rd9 mice at 3 and 6 days post injection (**Figure 4C**), (**B**) WT mice at 10 and 13 days post injection (**Figure 4E**).

frontiers
